# Toward Highly Dispersed Mesoporous Bioactive Glass Nanoparticles With High Cu Concentration Using Cu/Ascorbic Acid Complex as Precursor

**DOI:** 10.3389/fchem.2019.00497

**Published:** 2019-07-16

**Authors:** Kai Zheng, Jeonil Kang, Bogdan Rutkowski, Magdalena Gawȩda, Jue Zhang, You Wang, Niklas Founier, Maciej Sitarz, Nicola Taccardi, Aldo R. Boccaccini

**Affiliations:** ^1^Institute of Biomaterials, University of Erlangen-Nuremberg, Erlangen, Germany; ^2^Faculty of Metals Engineering and Industrial Computer Science, AGH University of Science and Technology, Kraków, Poland; ^3^Faculty of Materials Science and Ceramics, AGH University of Science and Technology, Kraków, Poland; ^4^Department of Bone and Joint Surgery, School of Medicine, Renji Hospital, Shanghai Jiao Tong University, Shanghai, China; ^5^Institute of Chemical Reaction Engineering, University of Erlangen-Nuremberg, Erlangen, Germany

**Keywords:** bioactive particles, sol-gel synthesis, mesoporous structure, copper, biomedical applications

## Abstract

Copper (Cu) ions have a variety of advantageous biological functionalities, such as proangiogenic and bactericidal activities. Given the intrinsic biodegradability and biocompatibility, silicate-based mesoporous bioactive glass nanoparticles (MBGNs) are considered as promising platforms for the delivery of Cu ions. However, effective incorporation of Cu into MBGNs still faces challenges, e.g., particle aggregation, the formation of insoluble crystalline Cu-based nanoparticles, and a low loading amount of Cu. We report a novel method to synthesize chemically homogenous and highly dispersed Cu-containing MBGNs (Cu-MBGNs) with tunable Cu concentration by using ascorbic acid/Cu complexes as the precursor of Cu in a microemulsion-assisted sol-gel approach. Cu-MBGNs exhibited a sphere-like shape with a particle size between 100 and 300 nm while their pore size varied from 2 to 10 nm. The inclusion of Cu, regardless of the incorporated concentration, did not significantly affect the morphology of particles. ICP-AES results indicated that the concentration of Cu in the particles could be conveniently tuned from 0 to ~6 mol% by controlling the amount of ascorbic acid/Cu complexes added, while the formation of crystalline Cu-based nanoparticles was avoided. The amorphous feature of Cu-MBGNs was proved by XRD, while the predominant oxidation state of Cu was evidenced to be Cu^2+^ by XPS. The incorporation of Cu did not inhibit the apatite-forming ability (bioactivity) of the particles in contact with simulated body fluid. Cu-MBGNs exhibited the capability of releasing Cu, Si, and Ca ions over time in the physiological fluid. The concentration of released Cu ions could be controlled by selecting specific Cu-MBGNs of different Cu contents. The dissolution products of most Cu-MBGNs at the dosage of 1, 0.1, and 0.01 mg/mL did not exhibit cytotoxicity, while only 7Cu-MBGN was cytotoxic at the dosage of 1 mg/mL. This study provided a feasible strategy to synthesize highly dispersed amorphous Cu-MBGNs with high Cu concentrations for biomedical applications. The particles exhibit great potential as building blocks for developing composite 3D scaffolds, coatings, and drug carriers, particularly when a large amount of particles incorporated may compromise the properties of (polymer) matrix materials while a relatively high concentration of released Cu ions is still required.

## Introduction

Mesoporous bioactive glass nanoparticles (MBGNs) are preferable biomaterials in a variety of biomedical applications (e.g., bone regeneration, drug delivery), due to their tailorable morphology (e.g., size, pore structure) and favorable physicochemical properties (e.g., bone bonding ability, biodegradability) (Vichery and Nedelec, 2016; Zheng and Boccaccini, 2017). Their ionic dissolution products, depending on the composition and concentration, can induce a series of beneficial biological responses toward enhanced therapeutic activity (Hoppe et al., 2011). Particularly, incorporation of metal ions in the glass composition, even at a low dose, can lead to promoted functionalities (Hoppe et al., 2011). For instance, the inclusion of silver (Ag) was able to enhance the antibacterial action of bioactive glass nanoparticles (BGN) (Kozon et al., 2016) while the presence of lithium (Li) could promote osteogenic activities (Zhang et al., 2018). Moreover, the physicochemical properties (e.g., mechanical properties, apatite-forming ability) of bioactive glasses (BG) can also be adjusted by incorporating specific metal elements (Kaur et al., 2014; Brauer, 2015).

BG have been used not only in regenerating hard tissues (e.g., bone, teeth) (Jones, 2015), but also in the repair of soft tissues (Miguez-Pacheco et al., 2015) and cancer treatment (Miola et al., 2018). For example, BG of some compositions have shown impressive ability to heal chronic skin wounds (Zhao et al., 2015; Naseri et al., 2017), mainly because of their antibacterial and proangiogenic properties (Broughton et al., 2006). Improvement in these properties can also benefit bone regeneration, especially for infected or large bone defects (Fernandes et al., 2017; Kargozar et al., 2018). Copper (Cu) ions have been well-known for their multifunctional biological activities (Li et al., 2016; Ryan et al., 2019). Depending on the concentration, Cu ions can exhibit bactericidal (Bari et al., 2017), proangiogenic (Wu et al., 2013), or pro-osteogenic activities (Burghardt et al., 2015). Given the amorphous characteristic of BG allowing a flexible adjustment in chemical composition, Cu-containing BG have been recognized as effective delivery platforms of Cu ions in comparison to bioceramics that must comply with specific stoichiometric ratios (Zhao et al., 2015; Wang et al., 2016), which have been applied as fillers in hydrogels (Wang et al., 2016) or coatings (Rau et al., 2019). Importantly, the release profiles of Cu ions from BG must be carefully controlled, because specific therapeutic effects are only initiated at a particular concentration window of Cu ions (Li et al., 2016; Ryan et al., 2019). For example, at a relatively low concentration, Cu ions tend to enhance angiogenesis (Romero-Sánchez et al., 2018), while they may cause toxicity toward healthy cells at a relatively high concentration (Milkovic et al., 2014). Being able to control the Cu concentration in a broad spectrum is thus critical to the application of Cu-containing BG for specific purposes (e.g., antibacterial or pro-angiogenic).

Copper-containing MBGNs (Cu-MBGNs) have been successfully synthesized using different sol-gel based strategies (Shi et al., 2015; Bari et al., 2017). However, particle aggregation and formation of CuO or Cu nanoparticles challenge the achievement of homogenous and dispersed MBGNs of a high Cu concentration (e.g., > 5 mol%). In conventional sol-gel approaches, Cu precursors (mostly Cu-based salts) are added during the sol-gel process, which may interrupt the particle formation/growth and cause aggregated particles with irregular shape and size (Brinker and Scherer, 1990). Careful control of the addition of these precursors (e.g., quantity) is thus necessary (Goh et al., 2014; Zheng et al., 2016b). Additionally, the effort of incorporating a relatively high content of Cu (e.g., > 5 mol%) in the particles may lead to the formation of Cu-based crystals (Bejarano et al., 2015; Zheng et al., 2017). In tissue regeneration strategies, MBGNs are preferably used as bioactive fillers incorporated into matrix materials (e.g., polymers, bioceramics or their combination) (El-Fiqi et al., 2015; Wang et al., 2016). Well-dispersed and chemically homogenous MBGNs are thus beneficial for the fabrication of MBGN-containing nanocomposites, which further improve the properties (e.g., mechanical properties) of the nanocomposites to a greater extent (Boccaccini et al., 2010; Leite and Mano, 2017). Moreover, the inclusion of Cu-MBGNs that have a relatively high concentration of Cu can reduce the amount of solid particles in the composites, if a certain amount of Cu ions should be achieved for specific therapeutic effects. In particular these fillers are preferred in the applications (e.g., composite hydrogels for 3D printing) where only a relatively low amount of particles can be incorporated (Leite and Mano, 2017; Yu et al., 2017).

In this study, we report a sol-gel based method to produce highly dispersed Cu-MBGNs of tunable Cu concentration by using a novel Cu/L-ascorbic acid complex as the precursor of Cu. The application of this precursor instead of Cu-based salts minimized the risk of particle aggregation and formation of Cu-based crystals. Furthermore, the concentration of Cu in particles could be conveniently tuned by controlling the amount of Cu precursor used. More importantly, a relatively high concentration of Cu (>5 mol%) could be incorporated into the particles without the expense of particle dispersity as well as chemical and morphological homogeneity. The synthesized Cu-MBGNs were also comprehensively characterized in terms of microstructure, chemical composition/structure, surface properties, ion release behavior, bioactivity, protein adsorption, and *in vitro* cytotoxicity, to explore their potential in biomedical applications.

## Materials and Methods

### Preparation of Cu/L-Ascorbic Acid Complex

Cu/L-ascorbic acid complex was prepared as reported in the literature (Xiong et al., 2011). Briefly, 50 mL of CuCl_2_ aqueous solution (0.2 M) was firstly prepared by dissolving CuCl_2_.2H_2_O (≥ 99.99%, Sigma-Aldrich) in 50 mL of deionized water. The CuCl_2_ aqueous solution was then heated at 80°C in an oil bath under magnetic stirring for 1 h before the dropwise addition of 50 mL of L-ascorbic acid (≥99%, Sigma-Aldrich) aqueous solution (0.4 M). The mixture was then allowed for a further reaction for 24 h at 80°C under stirring. After that, the mixture was centrifuged (Centrifuge 5430R, Eppendorf) at 7,000 rpm for 20 min to remove large aggregation and precipitates. The supernatant, composed of Cu/ascorbic acid complex suspension, was stored in a fridge (4°C) for further use.

### Synthesis of Cu-MBGNs

Cu-MBGNs were synthesized using a microemulsion-assisted sol-gel method as previously reported by Liang et al. (2015). Briefly, 0.56 g of cetrimonium bromide (CTAB, ≥ 97%, Sigma-Aldrich) was dissolved in 26 mL of deionized water under stirring. Eight milliliter of ethyl acetate (≥99.8%, Sigma-Aldrich) was added when CTAB was dissolved entirely. After stirring for 30 min, 5.6 mL of ammonia solution (1M, VWR) was added. After stirring for another 15 min, 2.88 mL of tetraethyl orthosilicate (TEOS, ≥99.0%, Sigma-Aldrich) and 1.83 g of calcium nitrate tetrahydrate (≥99.4%, VWR) were added sequentially at an interval of 30 min. After that, different volumes (0, 1, 5, 7, or 10 mL) of Cu/ascorbic acid complex were added to the mixture before further 4 h of reaction under stirring. The formed colloids were then collected by centrifugation at 7,197 rcf for 20 min, and washed twice with deionized water and once with ethanol (96%, VWR). The collected deposits were then dried at 60°C overnight before calcination at 700°C for 4 h with a heating rate of 2°C/min. The synthesized Cu-MBGNs were denoted as MBGN, 1Cu-MBGN, 5Cu-MBGN, 7Cu-MBGN, and 10Cu-MBGN, respectively, according to the volume of Cu precursors added.

As a comparison, Cu-MBGNs were also synthesized using copper nitrate hemi(pentahydrate), copper hydroxide, and copper chloride dihydrate (all purchased from Sigma-Aldrich) as the precursors of Cu. The resulting Cu-MBGNs were designated as CuN-MBGN, CuOH-MBGN, and CuCl-MBGN. The synthesis procedure was similar to the method described above. Only the Cu/L-ascorbic acid complex precursor was replaced with the above Cu precursors. The amount of these precursors was determined according to the designed nominal composition 70SiO_2_-25CaO-5CuO (in mol%), as 5 mol% of Cu in BG composition has been extensively reported in the literature (Goh et al., 2014; Shi et al., 2015; Bari et al., 2017). Additionally, such a Cu concentration could be incorporated into MBGNs by using the Cu/L-ascorbic acid complex as the precursor (the results are shown and discussed in the following sections).

### Physiochemical Characterization of Cu-MBGNs

The morphology of Cu-MBGNs was analyzed by using field emission scanning electron microscopy (FE-SEM, Auriga, Carl Zeiss) under an accelerating voltage of 2 kV. The microstructure of the particles was also observed by probe Cs-corrected scanning transmission electron microscopy (STEM, Titan^3^ G2 60–300, FEI) with high-angle annular dark field (HAADF) imaging. The chemical composition of Cu-MBGNs was characterized using energy dispersive X-ray (EDX) analysis (X-Max^N^, Oxford Instruments) during SEM observation. Additionally, inductively coupled plasma atomic emission spectroscopy (ICP-AES, SPECTRO CIROS-CCP spectrometer) was used to quantitatively determine the chemical composition of Cu-MBGNs. Briefly, the particles were first digested by using microwave heating, and 10 mL of concentrated HF/HNO_3_/HCl mixture in 1/1/3 volume ratio was used as the digestion medium. The resulting samples were then diluted to 100 mL with deionized water for the analysis.

The Zeta-potential of the samples was measured using a Zetasizer Nano ZS (Malvern Instruments, UK) instrument with a 4 mW HeNe laser (633 nm) and a light scattering detector positioned at 90°. The hydrodynamic size and polydispersity index (PDI) were examined under dynamic light scattering (DLS, Zetasizer Nano ZS) at 25°C, setting a minimum of 10 and a maximum of 100 runs per measurement. The measurements were performed in triplicate. For these measurements, the samples were dispersed in Dulbecco's phosphate-buffered saline (PBS, pH ~7.4, Gibco) at a concentration of 100 μg/mL.

Fourier transform infrared spectroscopy (FTIR, Nicolet 6700, Thermo Fisher Scientific) was also performed on Cu-MBGNs. The particles were mixed with KBr (VWR) and pressed to pellets at a weight ratio of 1:100 for the measurement. X-ray diffraction analysis (XRD) was conducted using X-ray diffractometer (Rigaku, MiniFlex 600, Japan) in a 2θ range of 20–80°. A step size of 0.020° with a dwell time of 1 s per step was used. The Brunauer-Emmett-Teller (BET) specific surface area (SSA) and pore size distribution of Cu-MBGNs were determined by using the nitrogen sorption analysis, conducted on a Micromeritics porosimeter (ASAP2460, Micrometrics Instrument). High-resolution X-ray photoelectron spectroscopy (XPS) spectra of the samples were recorded with a Thermo Scientific ESCALAB 250Xi spectrometer using monochromatic Al Kα X-rays under a vacuum of 5 × 10^−10^ Torr.

### *In vitro* Apatite Formation

The *in vitro* apatite-forming ability (bioactivity) of Cu-MBGNs was evaluated by soaking the particles in simulated body fluid (SBF) as reported by Kokubo and Takadama (2006). Briefly, the particles were soaked in SBF at a concentration of 1 mg/mL and kept in an incubator (KS 4000i control, IKA, Germany) at 37°C shaking at 90 rpm for up to 7 d. At predetermined time points, the particles were collected and rinsed with acetone before drying at 60°C overnight. The formation of apatite on the particles was then characterized by SEM, FTIR, and XRD.

### Protein Adsorption

Bovine serum albumin (BSA; Sigma-Aldrich) was used as the model protein to assess the protein adsorption capacity of MBGN and Cu-MBGNs. Quantification was carried out by using the colorimetric bicinchoninic acid (BCA; Thermo Fisher) assay. Briefly, the particles were soaked in BSA aqueous solution (500 μg/mL) at a concentration of 4 mg/mL at 37°C. At different time points, 50 μL of supernatant from each sample was removed and mixed with 1 mL BCA working solution. After a further 30 min of incubation at 37°C, the protein concentration in the supernatant was quantified by a UV-vis spectrophotometer (Specord^®^40, Analytik Jena, Germany) at 532 nm. The adsorbed protein values were calculated by subtracting the measured protein concentration in the supernatant from the initial concentration of protein solution (500 μg/mL).

### Ion Release of Cu-MBGNs

The ion release behavior of Cu-MBGNs was analyzed in Tris-HCl buffer (Trizma^®^ Pre-set crystals, Sigma-Aldrich, pH 7.4). Briefly, 10 mg of MBGN and Cu-MBGNs were soaked in 20 mL of Tris-HCl solution in an incubator at 37°C shaking at 90 rpm for up to 7 d. At each predetermined time point, the samples were centrifuged. Half of the supernatant (10 mL) was then collected and replenished with fresh 10 mL of Tris–HCl. The ionic concentration of the supernatant was analyzed by using ICP-AES.

### *In vitro* Cytotoxicity

The cytotoxicity of dissolution products of Cu-MBGNs was assessed by using a colorimetric (WST-8 based) assay. Mesenchymal stromal ST2 cells (DSMZ) derived from mouse bone marrow of BC8 mice were used. The cells were cultured at 37°C in a humidified atmosphere of 95% air and 5% CO_2_ in culture medium composed of RPMI 1640 medium (Gibco), 10 vol% fetal bovine serum (FBS, Sigma-Aldrich), and 1 vol% penicillin/streptomycin (Gibco). The cells were cultured to confluence in 75 cm^2^ culture flasks, harvested using Trypsin/EDTA (Gibco), and counted using a hemocytometer (Roth).

MBGN and Cu-MBGNs were soaked in the culture medium at a concentration of 1 mg/mL for 24 h. After the soaking, the supernatant was collected, while parts of them were diluted to 10% (v/v) and 1% (v/v) with culture medium. All these extracts were then placed in 24-well plates with ST2 cells at a density of 1 × 10^5^ cells/well. After incubation for 48 h, the culture medium was then removed from the wells and washed with PBS. Afterward, 0.25 mL of a WST medium (containing 1 vol% WST reagent and 99 vol% culture medium) was added and incubated for further 2 h. After that, 0.1 mL of the supernatant was transferred to a 96-well plate and spectrometrically measured using a microplate reader (Phomo, Anthos Mikrosysteme GmbH) at 450 nm. Each experiment was carried out in triplicate. The difference between the samples investigated was analyzed by one-way analysis of variance (ANOVA). The level of the statistical significance was defined as *p* < 0.05 (Origin 8.1G, Origin Lab Corporations).

## Results and Discussion

### Synthesis and Characterization of Cu-MBGNs

**Figure 1:**
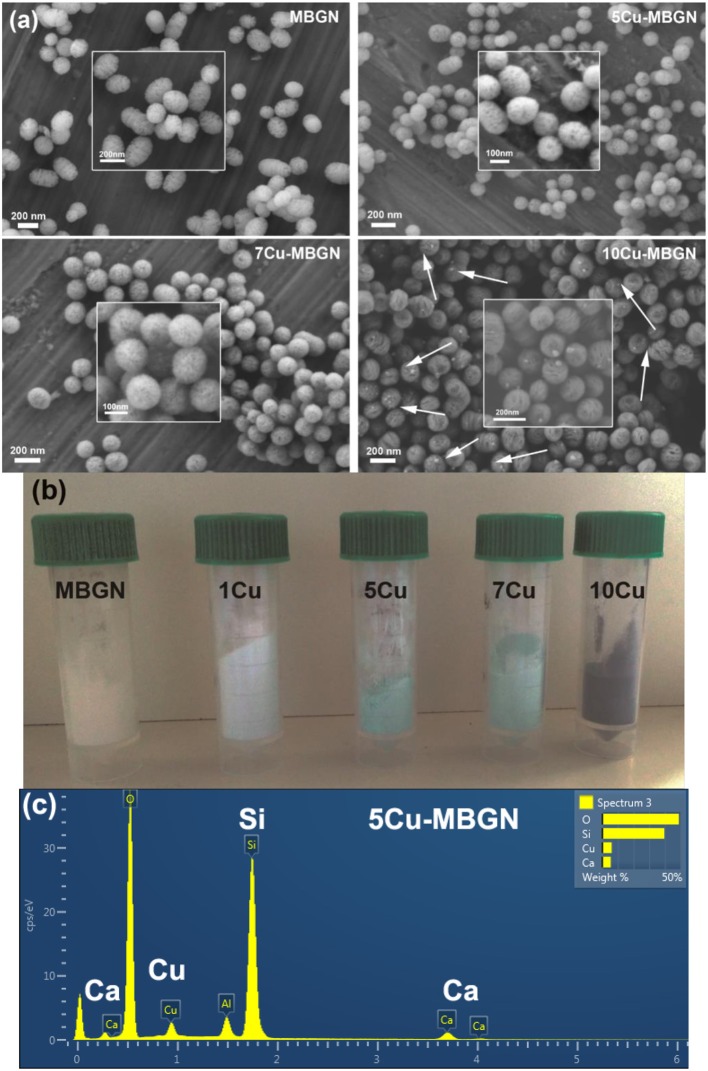
**(a)** Representative SEM images of particles with inserted high magnification images. Arrows indicate the formed CuO nanoparticles; **(b)** An optical image of MBGN and Cu-MBGNs; **(c)** A representative EDS spectrum of 5Cu-MBGN shows the presence of Si, Ca, and Cu. The peak of Al is detected from the substrate of the conductive Aluminum tape.

Figure 1a shows representative SEM images of MBGN and 5Cu-, 7Cu-, and 10Cu-MBGN, in which all the particles show a sphere-like shape with visible mesopores on the surface. The size of all the particles appeared to be in the range of 100 to 300 nm. The morphology is in good agreement with that of particles synthesized by using similar approaches reported in the literature (Liang et al., 2015; Nawaz et al., 2018). 1Cu-MBGN exhibited similar morphology to other Cu-MBGNs (Figure S1). The addition of Cu/L-ascorbic acid complex regardless of volumes did not significantly affect the morphology of the resulting particles. However, smaller nanoparticles clustering on larger mesoporous particles could be observed in 10Cu-MBGN (Figure 1a) while no such particles were seen in other Cu-MBGNs and MBGN. Figure 1b shows an optical image of all synthesized nanoparticles. The color of the particles changed from whitish to darker blue with increasing the amount of Cu precursors added, suggesting the increased content of incorporated Cu ions. Figure 1c shows the representative EDS spectrum of 5Cu-MBGN that confirmed the presence of Si, Ca, and Cu in the particles. The EDS results (Figure S2) of other particles also proved the presence of Si, Ca, and Cu ions. Table 1 shows the chemical composition of MBGN and Cu-MBGNs determined by using the EDS results. The chemical composition of MBGN was determined as ~84.6SiO_2_-15.4CaO (mol%), which was different from their nominal composition (70SiO_2_-30CaO, mol%). Such a composition gap between theoretical and actual compositions of nanoscale BG has been usually observed, due to the mechanism of particle formation in base-catalyzed sol-gel processes (Liang et al., 2015; Zheng et al., 2016b; Zheng and Boccaccini, 2017). As expected, the molar concentration of Cu in Cu-MBGNs increased with increasing the amount of Cu precursor added, indicating the tailorability of Cu concentration in the particles. We also quantitatively analyzed the chemical composition of the particles by using ICP-AES analysis. Similarly, the concentration of Cu in the particles increased with the amount of Cu precursor added. Additionally, the determined concentrations of Ca and Cu in the particles were slightly lower than those determined in the EDS analysis, which was usually observed in the comparison between EDS and ICP-AES results for determining the chemical composition of BG nanoparticles (Greasley et al., 2016). It should be pointed out that 10Cu-MBGN were not analyzed by ICP-AES as they contained undesired crystalline Cu-based nanoparticles (Figure 1a). Both ICP-AES and EDS results confirmed that a relatively high concentration of Cu (>5 mol%) could be successfully incorporated into MBGN.

**Table 1:** Chemical compositions (mol%) of MBGN and Cu-MBGNs determined from the EDS and ICP-AES results.

**Samples**		**SiO_**2**_**	**CaO**	**CuO**
MBGN	EDS	84.6 ± 0.9	15.4 ± 0.8	n.a.
	ICP-AES	85.8 ± 1.1	14.2 ± 0.9	n.a.
1Cu-MBGN	EDS	84.6 ± 1.3	13.6 ± 0.4	1.8 ± 0.2
	ICP-AES	86.0 ± 1.7	13.1 ± 0.1	0.9 ± 0.1
5Cu-MBGN	EDS	83.1 ± 1.4	11.1 ± 0.3	5.8 ± 0.3
	ICP-AES	85.4 ± 0.6	10.2 ± 0.3	4.4 ± 0.4
7Cu-MBGN	EDS	81.2 ± 1.7	11.6 ± 0.4	7.2 ± 0.5
	ICP-AES	84.7 ± 1.1	9.2 ± 0.5	6.1 ± 0.3
10Cu-MBGN	EDS	79.6 ± 1.7	9.6 ± 0.7	10.8 ± 1.5

**Figure 2:**
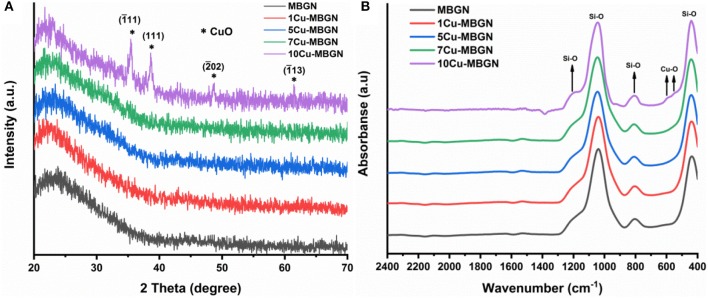
**(A)** XRD patterns and **(B)** FTIR spectra of MBGN and Cu-MBGNs. ^*^Indicates the crystallographic planes of CuO.

**Figure 3:**
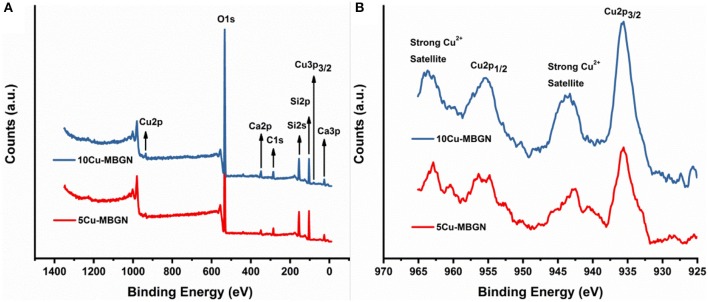
XPS spectra of 5Cu-MBGN and 10Cu-MBGN. **(A)** XPS survey scan and **(B)** high-resolution scan of Cu 2p3/2 and Cu 2p1/2 peaks.

We further analyzed the crystallization and chemical structure of the synthesized particles. Figure 2A shows the XRD patterns of MBGN and Cu-MBGNs. As expected, MBGN showed a typical XRD pattern of amorphous silicate materials, in which only a broad band located at 2θ = 23° corresponded to amorphous silicate could be observed (Bari et al., 2017). Similarly, only this broadband could be found in the XRD spectra of 1Cu-MBGN, 5Cu-MBGN, and 7Cu-MBGN, indicating that the incorporated Cu did not form crystals or the amount of the crystals was lower than the XRD detection level. However, diffraction peaks located at 2θ = ~35.5°, 38.6°, 48.5°, and 61.5° assigned to $\left(\bar{1}11\right)$, (111), $\left(\bar{2}02\right)$, and $\left(\bar{1}13\right)$ crystallographic planes of CuO (JCPDS 05-0661) (Thekkae Padil and Cerník, 2013) could be observed in 10Cu-MBGN, which proved that the nanoparticles observed in SEM image of 10Cu-MBGN are CuO crystals (Figure 1a). The presence of the CuO crystals could also explain the higher concentration of Cu in 10Cu-MBGN (Table 1). Figure 2B shows the FTIR spectra of MBGN and Cu-MBGNs, where all particles showed similar characteristic bands to silicate glasses. Two bands located at ~440 and 812 cm^−1^ could be assigned to Si–O–Si bending and symmetric stretching vibrations, respectively, while the bands located in the range 1,299–900 cm^−1^ could be assigned to asymmetric Si-O-Si (bridging bonds) and Si-O-(non-bridging bonds) vibrations (Sitarz et al., 2005; Aguiar et al., 2009; Wajda and Sitarz, 2018). The presence of these bands indicated that [SiO_4_] tetrahedrons had formed to build the glass network of the particles (Aguiar et al., 2009). In the spectrum of 10Cu-MBGN, two bands located at ~545 and 596 cm^−1^ could be observed, which are probably related to the stretching Cu–O in CuO crystals (Thekkae Padil and Cerník, 2013). No significant changes were found in the FTIR spectra after the incorporation of different concentrations of Cu, while only the trace of CuO in 10Cu-MBGN was observed in the spectrum. We also confirmed the presence of Cu species and identified the primary oxidization state of Cu in Cu-MBGNs by using XPS. 5Cu-MBGN and 10Cu-MBGN were selected as the model Cu-MBGNs for this characterization. The XPS survey scans (Figure 3A) showed characteristic binding energy peaks of carbon, oxygen, silicon, calcium, and copper, which were consistent with the EDS results. The Cu2p core level spectra of both particles (Figure 3B) represent four peaks. The peaks located at approximately 935 and 955 eV were related to Cu 2p_3/2_ and Cu 2p_1/2_, respectively (Kim et al., 2006). The strong satellites recorded in both samples confirmed that the main oxidation state of Cu was Cu^2+^ (Kim et al., 2006).

We also evaluated the surface charge and dispersity of MBGN and Cu-MBGNs in physiological solution PBS, considering the essential roles of these properties in the applications as drug/ion carriers and fillers in composites. All particles exhibited negatively surface charge in PBS as indicated by their negative zeta potential values (Table 2). Notably, the zeta potential values of the particles became closer to zero with increasing Cu content, probably due to the elevated amount of positively charged Cu ions in Cu-MBGNs, which is in good agreement with results in the literature (Zheng et al., 2017). The hydrodynamic size of all particles was similar to each other, which seemed not affected by the inclusion of Cu. All particles also exhibited relatively low PDI values, which suggested that these particles could form stable suspensions in aqueous solution for composite fabrication (e.g., coatings, biopolymer-based scaffolds). However, the stability and dispersity of these particles might be compromised due to the presence of proteins or salts in *in vivo* conditions. Further surface modification is thus required to enhance the stability of Cu-MBGNs for *in vivo* drug delivery (Xue et al., 2019).

**Table 2:** Physicochemical characterization results of MBGN and Cu-MBGNs.

**Samples**	**Zeta potential (mV)**	**DLS size (nm)**	**PDI**	**SSA (m^**2**^/g)**	**Pore volume (cm^**3**^/g)**
MBGN	−27 ± 2	230 ± 19	0.151	319	0.39
1Cu-MBGN	−19 ± 1	223 ± 16	0.132	298	0.42
5Cu-MBGN	−16 ± 2	242 ± 26	0.113	317	0.43
7Cu-MBGN	−17 ± 1	236 ± 23	0.108	342	0.46

The results indicated that the concentration of Cu in Cu-MBGNs could be conveniently tuned from 0 to ~6 mol% without causing the formation of Cu-based crystalline nanoparticles. Moreover, the morphology and dispersity of the particles were not significantly influenced by the inclusion of Cu. In conventional sol-gel based BG synthesis, CuO nanoparticles or other Cu-based crystals tend to form, particularly when a relatively high Cu concentration (e.g., >5 mol%) is attempted (Bejarano et al., 2015; Mabrouk et al., 2019). Cu salts (e.g., copper nitrate, copper chloride) are usually used as the Cu precursor because they are cost-effective and highly dissoluble in water or alcohol solvents. However, the introduction of these salts into sol-gel processes can alter the reaction conditions (e.g., ionic strength, pH, particle stability), which can cause particle aggregation and irregular shape and size of particles (Brinker and Scherer, 1990). Therefore, the addition of salts during the synthesis of nanoscale BG must be carefully controlled to achieve homogeneous and dispersed particles (Zheng et al., 2017). With the assistance of other techniques (e.g., ultrasonication) (Bari et al., 2017) or additional acid catalysts (Goh et al., 2014), a higher concentration of Cu could be incorporated into MBGNs without causing the formation of Cu-based crystals, but the uniformity in size and dispersity could be compromised. In this study, we used a novel Cu/L-ascorbic acid complex precursor that has been previously used to synthesize Cu nanoparticles (Xiong et al., 2011). The presence of antioxidant agent L-ascorbic acid could prevent Cu from being oxidized to copper oxide. Therefore, this Cu/L-ascorbic acid complex precursor is stable under ambient conditions (Xiong et al., 2011). During the synthesis of Cu-MBGNs, the stable Cu/L-ascorbic acid complex could be converted to ionic Cu (Cu^2+^ or Cu^1+^) in the presence of ammonia (Ji et al., 2012). This ionic Cu based complex could be firmly adsorbed by mesoporous silica-based nanoparticles, which enhanced the adsorbed amount of Cu ions in MBGNs (Ren et al., 2015). Importantly, the presence of L-ascorbic acid prevented the formation of precipitates of copper (II) hydroxide that could be converted to CuO during high-temperature treatment (Hathaway and Tomlinson, 1970).

**Figure 4:**
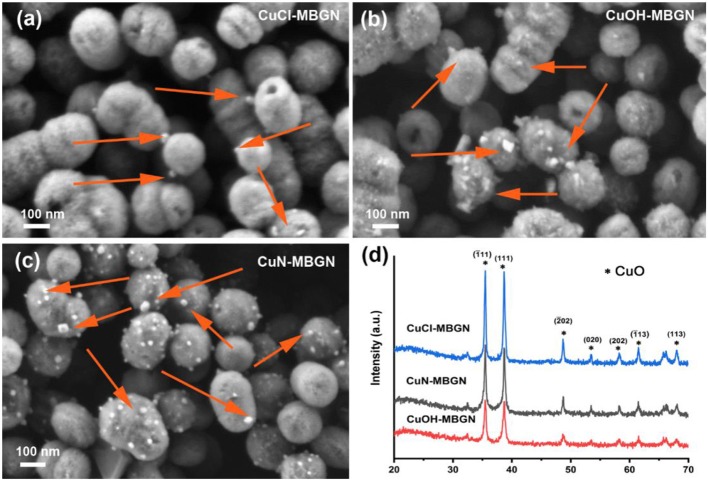
SEM images of **(a)** CuCl-MBGN, **(b)** CuOH-MBGN, and **(c)** CuN-MBGN. Arrows indicate CuO nanoparticles. **(d)** XRD patterns of CuN-MBGN, CuOH-MBGN, and CuCl-MBGN. ^*^Indicates the crystallographic planes of CuO.

As a comparison, we also synthesized Cu-MBGNs by using conventional Cu precursors (i.e., copper nitrate, copper chloride, and copper hydroxide) in this microemulsion-assisted sol-gel approach. A nominal concentration of 5 mol% Cu was designed for the synthesis, as this concentration could be obtained by using the Cu/L-ascorbic acid complex precursor (Table 1). Figures 4a–c show SEM images of CuN-MBGN, CuOH-MBGN, and CuCl-MBGN. The nanoparticles exhibited similar morphology and shape to those synthesized using the Cu/L-ascorbic acid complex. However, smaller nanoparticles could be observed in all of these mesoporous particles, which appeared to be similar to CuO particles in 10Cu-MBGN (Figure 1a). XRD results (Figure 4d) confirmed that the formed smaller nanoparticles were CuO crystals (JCPDS 05-0661) (Thekkae Padil and Cerník, 2013). The results showed that chemically homogeneous Cu-MBGNs with 5 mol% Cu or higher could be hardly synthesized by using conventional Cu precursors in base-catalyzed sol-gel processes. The use of this novel Cu/L-ascorbic acid complex precursor was therefore the key step to obtain highly dispersed and chemically homogeneous Cu-MBGNs of high Cu concentration. The uniformity in size and shape, high dispersity, as well as the tailorable Cu concentration, particularly benefit the applications of Cu-MBGNs as fillers in coatings or scaffolds (Pontremoli et al., 2018; Yang et al., 2018; Zheng et al., 2018). This precursor is also expected to be able to increase the Cu content in non-porous spherical BGN synthesized by the well-established Stöber method (Zheng et al., 2017). Therefore, 10Cu-MBGN were not discussed in the following sections, as they contained crystalline CuO that was not the main focus in this study.

### Mesoporous Structure of Cu-MBGNs

**Figure 5:**
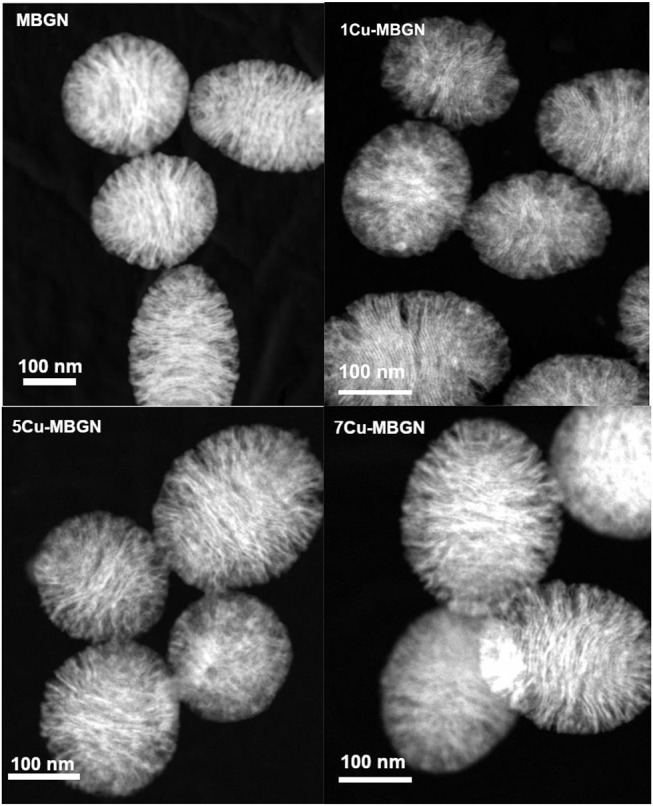
STEM-HAADF images of MBGN, 1Cu-MBGN, 5Cu-MBGN, and 7Cu-MBGN reveal the mesoporous structure of particles.

In addition to SEM images (Figure 1a), STEM images further confirmed the presence of mesopores on MBGN and Cu-MBGNs (Figure 5). All the particles exhibited a dendritic pore shape which was consistent with that of the mesoporous nanoparticles reported in the literature (Liang et al., 2015), indicating that the addition of Cu/L-ascorbic acid complex did not significantly affect the self-assembly of mesophases during the reaction. Also, the pore size seems not homogenous, which is commonly observed in microemulsion-assisted mesoporous particles (Liang et al., 2015; Nawaz et al., 2018). No additional smaller Cu-based nanoparticles could be seen in STEM images, which is in agreement with the observation in SEM images and XRD results.

**Figure 6:**
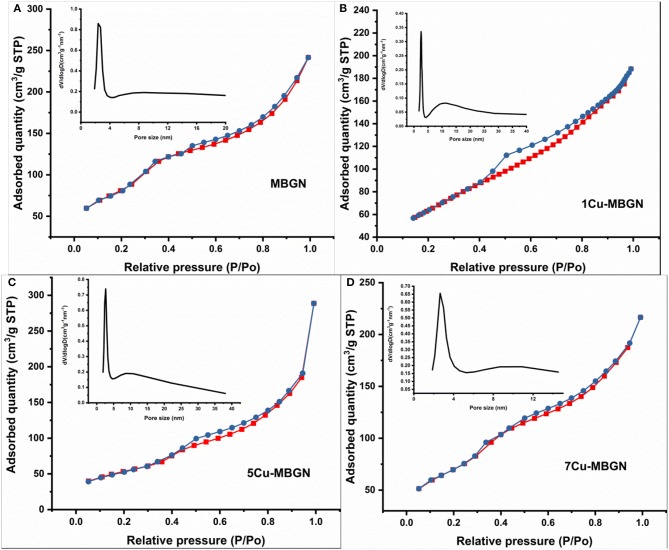
N_2_ adsorption-desorption isotherm and inserted pore size distribution of **(A)** MBGN, **(B)** 1Cu-MBGN, **(C)** 5Cu-MBGN, and **(D)** 7Cu-MBGN.

We further confirmed the mesoporous structure of Cu-MBGNs by using the nitrogen sorption analysis. Figure 6 shows the N_2_ adsorption-desorption isotherm of MBGN, 1Cu-MBGN, 5Cu-MBGN, and 7Cu-MBGN. All of them exhibited type IV isotherms as defined by IUPAC (Brunauer et al., 1940), which is a typical isotherm for mesoporous materials. All the particles had a narrow pore size distribution centered at ~2.5 nm, but a wide pore distribution starting at ~8 nm could also be observed, which was consistent with the observation (presence of dendritic pores) in STEM images (Figure 5). All the particles exhibited large SSA and pore volume (Table 2), which is expected to facilitate protein adsorption or drug loading. Moreover, the presence of Cu in the particles did not significantly influence the SSA. The pore volume, another critical parameter related to drug loading/protein adsorption, was not significantly affected by the incorporation of Cu (Table 2).

### *In vitro* Bioactivity, Dissolution, Protein Adsorption, and Cytotoxicity

**Figure 7:**
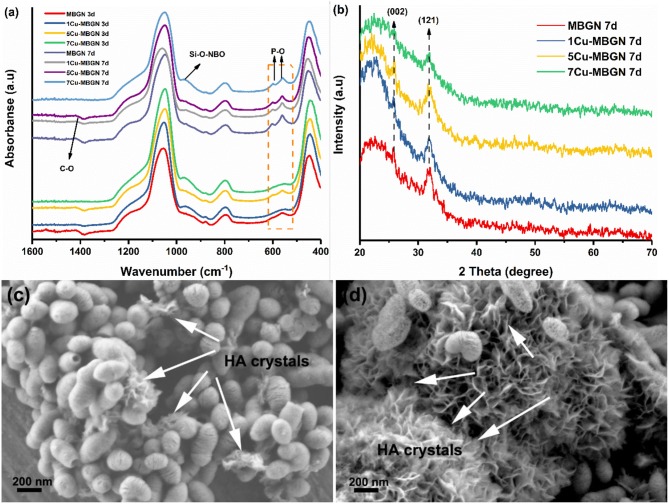
Evaluation of *in vitro* apatite formation on MBGN and Cu-MBGNs. **(a)** FTIR spectra of MBGN and Cu-MBGNs after immersion in SBF for 3 and 7 d; **(b)** XRD patterns of these particles after immersion in SBF for 7 d; Representative SEM images of 5Cu-MBGN after immersion in SBF for 3 d **(c)** and 7 d **(d)**. Arrows indicate HA crystals. Representative SEM images of MBGN, 1Cu-MBGN, and 7Cu-MBGN after immersion in SBF shown in Figure S3.

Figure 7a shows FTIR spectra of MBGN and Cu-MBGNs after immersion in SBF for up to 7 d. Two new bands located at ~560 and ~604 cm^−1^ could be observed in the FTIR spectra of the particles after immersion in SBF for 3 d, which could be assigned to the P-O bending vibrations in P${\text{O}}_{3}^{-4}$ (Jastrzbski et al., 2011; Zheng et al., 2015). These bands became more evident after 7 d of immersion in SBF. The observation of such bands suggested the formation of crystalline calcium phosphate phases. Additionally, a small band located at ~1,420 cm^−1^ was also formed during the immersion in SBF, which could be related to the formation of carbonates (Cerruti and Morterra, 2004), suggesting that the formed calcium phosphate phases could be carbonate hydroxyapatite (HCA). A slight band at ~966 cm^−1^, associated to the Si–O with one non-bridging oxygen (Si–O–NBO) per [SiO_4_] tetrahedron, appeared after 3 d immersion in SBF, which suggests the exchange of network modifying ions of the glass with the ions in SBF and the dissolution of the particles (Serra et al., 2003). Further, the characteristic bands of silicate located at ~440, 812, and 1,043 cm^−1^ could still be observable after immersion in SBF for 7 d, indicating the preservation of silicate structure of the particles. XRD patterns (Figure 7b) of the particles after immersion in SBF for 7 d confirmed the formation of crystalline hydroxyapatite (HA). Diffraction peaks located at approximately 2θ = 26.9° and 32° related to HA crystals (JCPD 84-1998) (Shih et al., 2015) could be observed in MBGN, 1Cu-MBGN, and 5Cu-MBGN after immersion in SBF for 7 d, but only the diffraction peak located at 32° could be found in 7Cu-MBGN, which suggested the lower crystallinity of formed apatite in 7Cu-MBGN. However, the crystallinity of all the particles after immersion in SBF for 7 d as determined by XRD patterns was not high, which could be due to the presence of a large quantity of remaining amorphous BG particles. Figures 7c,d show representative SEM images of 5Cu-MBGN after immersion in SBF for 3 and 7 d, respectively. Needle-like crystals, characteristic morphology of HA crystals forming on BG surfaces after exposure to SBF (Zheng et al., 2016a), could be observed on 5Cu-MBGN after immersion in SBF for 3 d. With increasing immersion time in SBF, a larger quantity of needle-like apatite crystals formed. Although the mesoporous particles started to dissolve after immersion in SBF, as evidenced by the enlarged pores (Figure 7d), a large amount of spherical particles could still be seen, which is consistent with the results in FTIR and XRD. Similar needle-like HA crystals could also be found in MBGN, 1Cu-MBGN, and 7Cu-MBGN after immersion in SBF (Figure S3). However, no significant difference could be distinguished in the apatite formation ability of all the particles. The influence of Cu in BG on the apatite-forming ability of glasses is complex. Substitution of Ca with Cu in BG may enhance apatite formation, but other components in BG and the amount of substituted Cu should also be considered (Bejarano et al., 2015). In our previous work, we have shown that approximately 2 mol% substitution of Ca with Cu could accelerate the formation of apatite on Cu-containing BGN (Zheng et al., 2017). However, it is challenging to conclude how the presence of Cu in MBGN influences the apatite formation based on the current results. Nevertheless, the results proved that MBGN and Cu-MBGNs could induce rapid apatite formation during the immersion in SBF, which indicated their high bioactivity and potential for bone-related applications.

**Figure 8:**
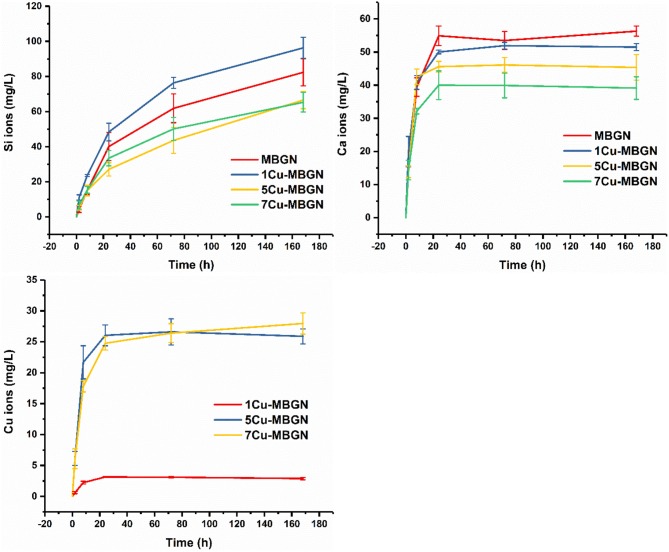
Ion release profiles of MBGN, 1Cu-MBGN, 5Cu-MBGN, and 7Cu-MBGN in Tris-HCl solution.

It is known that the ionic dissolution products of BG can induce beneficial biological responses (e.g., pro-osteogenic and proangiogenic activities) for tissue regeneration (Hoppe et al., 2011). The achievement of these desired effects is dependent on the concentration and type of ions. We evaluated the ion release of Cu-MBGNs to understand the dissolution and release profile of the particles. Figure 8 shows the release profiles of ionic Si, Ca, and Cu from the particles for up to 7 d in Tris-HCl. All MBGN and Cu-MBGNs exhibited a similar release profile of Si, in which a continuous and sustained release was observed, indicating the continuous dissolution of silicate particles over time. After 7 d of immersion, MBGN and 1Cu-MBGN released out a significantly high concentration of Si ions than 5Cu-MBGN and 7Cu-MBGN, which was probably related to their intrinsically higher content of SiO_2_. However, 1Cu-MBGN released more Si ions than MBGN, even though they had comparable contents of SiO_2_ in the composition. This phenomenon suggested that incorporation of a relatively low amount of CuO might enhance the dissolution of silicate MBGN. Furthermore, all particles exhibited a burst release of Ca ions within 8 h, while the concentration of released Ca ions appeared to be stable after 24 h of release for all particles. We calculated the percentage of released Ca ions in relation to the actual amount of Ca in the particles that was determined by ICP-AES (Figure S4). Notably, almost all Ca ions have been released out from the particles after 24 h of immersion in Tris-HCl. Similarly, a burst release of Cu ions within 8 h was also observed for Cu-MBGNs, which is in agreement with literature reports (Hoppe et al., 2013; Bari et al., 2017). Notably, most Cu ions in 5Cu-MBGN were released out after 24 h immersion in Tris-HCl, while 1Cu-MBGN and 7Cu-MBGN still contained unreleased Cu ions (Figure S4). As expected, MBGN released out the highest concentration of Ca ions while 7Cu-MBGN did the lowest, given their intrinsic chemical composition (Table 1). The fast release of Ca and Cu ions suggests that these ions were mainly located at the outer layers of particles, as these ions only diffused into the inner parts of particles from the surface during the heat treatment (Zheng and Boccaccini, 2017). It should be pointed out that BG could release ions faster in Tris-HCl in comparison to that in other physiological fluids (e.g., SBF, PBS), probably due to no formation of calcium phosphate species that could inhibit further dissolution of BG in Tris-HCl (Varila et al., 2012). Altogether, the results showed that both MBGN and Cu-MBGNs could dissolve in physiological fluids and release bioactive ions during their dissolution. Furthermore, various Cu-MBGNs exhibited different ion release behavior in terms of ion concentration, enabling the control of Cu ion release for specific applications, e.g., a relatively low concentration of Cu ions for angiogenesis while a relatively high one for antibacterial activity, by selecting suitable Cu-containing particles in different scenarios.

**Figure 9:**
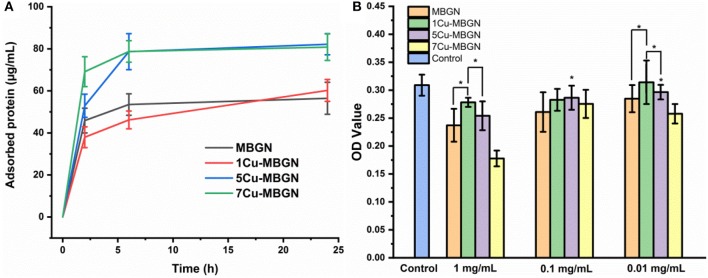
**(A)** BSA adsorption on MBGN and Cu-MBGNs over time. **(B)** WST assay results of the dissolution products of MBGN and Cu-MBGNs in culture with mesenchymal stromal ST2 cells for 48 h. All statistical significance was shown in comparison to the control unless indicated (^*^p < 0.05).

When BG encounter protein-containing biological fluids (e.g., blood plasma, interstitial fluids) *in vivo*, protein adsorption is the very first event taking place on BG surfaces (Rabe et al., 2011). The adsorbed protein layer can influence the cellular activities of BG (e.g., cell adhesion and differentiation) and the biological fate of BG nanoparticles (Zheng et al., 2019). We evaluated the BSA adsorption on Cu-MBGNs over time to understand the protein adsorption capability of particles and the influence of Cu incorporation on the adsorption behavior. It appeared that the incorporation of Cu could enhance protein adsorption, as 5Cu-MBGN and 7Cu-MBGN adsorbed a larger amount of BSA than MBGN and 1Cu-MBGN (Figure 9A). The less negatively charged surface of 5Cu-MBGN and 7Cu-MBGN (Table 2) could facilitate the adsorption of BSA. In a solution with low ionic strength (in this case, ultrapure water), electrostatic interactions play a major role in driving protein adsorption (Rabe et al., 2011). As BSA is also negatively charged in water, less negatively charged 5Cu-MBGN and 7Cu-MBGN could thus adsorb more BSA. Such an enhancement in protein adsorption could also be related to the incorporation of Cu enlarging SSA of particles (Table 2). Generally, particles with higher SSA could adsorb a larger amount of proteins (Zheng et al., 2019). Notably, a limited amount of BSA could be adsorbed on the particles after 6 h, which suggested the achievement of protein adsorption equilibrium on the particles. Protein adsorption is a rather complex process, as many factors can affect it, from the surface properties of materials (e.g., surface charge, surface area) to the environmental conditions such as pH and temperature (Rabe et al., 2011). Given the bioreactive surface of BG, even more events (e.g., biomineralization, surface degradation) are involved in the process of protein adsorption on BG surfaces (Zheng et al., 2019). In this study, we did not take account of the influence of apatite formation that also plays a significant role in protein adsorption. A more comprehensive and systematic investigation is still required to understand the influence of Cu incorporation on protein adsorption *in vivo*.

Finally, we evaluated the cytotoxic effects of dissolution products of MBGN and Cu-MBGNs against mesenchymal stromal ST2 cells. At a relatively high dosage of 1 mg/mL (Figure 9B), compared to the control group (particle-free culture), all the particles induced lower cell viability as indicated by the reduced metabolic activity. However, only 7Cu-MBGN exhibited a significantly reduced cell viability (<80% compared to the control), which could be due to the highest concentration of released Cu ions. Notably, MBGN reduced the viability of cells to a greater extent than 1Cu-MBGN and 5Cu-MBGN at this dosage, probably due to their higher concentration of released Ca ions inducing greater pH increase (Ciraldo et al., 2018). However, the relative cell viability in the sample groups (excluding 7Cu-MBGN) was still higher than 80% compared to the control group, indicating the non-cytotoxicity of the particles at 1 mg/mL. By lowering the concentration of the dissolution products, the cell viability increased in all groups, while no significant difference between the concentrations of 0.1 and 0.01 mg/mL was observed. Compared to the control, the cell viability of all samples was higher than 80%, suggesting the non-cytotoxicity of particles at the dosage of 0.1 and 0.01 mg/mL. Our results showed that the cytotoxicity of Cu-MBGNs was related to the concentration of Cu ions as well as the dosage of particles applied. It has been reported that Cu could exert angiogenic and osteogenic effects at a relatively low concentration (Romero-Sánchez et al., 2018; Ryan et al., 2019) while a relatively high one could cause toxicity toward healthy cells (Milkovic et al., 2014) or bacteria. Therefore, the release of Cu ions (e.g., concentration, release profile) must be tunable and controllable according to the end applications. Owing to the distinctive ion release behavior of various Cu-MBGNs, the control of Cu ion release could be achieved by selecting specific Cu-MBGNs. For example, even though 7Cu-MBGN showed cytotoxicity at the high dosage of 1 mg/mL, they could still be used as fillers in composites, as in these situations the concentration of released Cu ions could be tuned by controlling the amount of particles used, through which an appropriate release profile could be achieved for specific purposes (e.g., pro-angiogenic activity). Moreover, Cu-MBGNs of a high Cu concentration (e.g., 7Cu-MBGN) are preferred in applications where only a relatively low amount of particles can be incorporated (e.g., composite hydrogels for 3D printing).

## Conclusions

We successfully synthesized Cu-containing mesoporous bioactive glass nanoparticles (Cu-MBGNs) with tunable Cu concentration by using Cu/ascorbic acid complex as the precursor of Cu in a microemulsion-assisted sol-gel approach. A high concentration of Cu (up to ~6 mol%) could be incorporated into MBGNs without causing the formation of Cu-based crystalline particles. Additionally, the morphology, dispersity, and mesoporous structure of particles were preserved after the incorporation of Cu. The synthesized Cu-MBGNs also exhibited high bioactivity (fast apatite formation in SBF) and could release biologically active ions during the dissolution. The cytotoxicity of Cu-MBGNs was related to the dosage of particles and the concentration of Cu in the composition. Only 7Cu-MBGN showed cytotoxicity at the dosage of 1 mg/mL, while other particles were non-cytotoxic at the tested dosages. The tunable level of Cu concentration and advantageous morphology enable Cu-MBGNs to be used for different purposes (e.g., antibacterial or angiogenic properties). The synthesized particles thus show great potential as bioactive fillers, coatings, or drug delivery carriers in a variety of biomedical applications including bone regeneration and wound healing.

## Data Availability

All datasets generated for this study are included in the manuscript and/or the Supplementary Files.

## Author Contributions

KZ conceived the initial idea and designed the experiments with AB and also analyzed the results and wrote the paper. JK carried out most of the experiments and analyzed the results. BR, MG, JZ, YW, NF, MS, and NT carried out part of the characterization and provided analysis of the data. AB conceived the initial idea and contributed to the discussion of results. All authors read the manuscript and provided inputs.

### Conflict of Interest Statement

The authors declare that the research was conducted in the absence of any commercial or financial relationships that could be construed as a potential conflict of interest.
