# Supplementary material for: Toward Highly Dispersed Mesoporous Bioactive Glass Nanoparticles With High Cu Concentration Using Cu/Ascorbic Acid Complex as Precursor
Source: Front Chem. 2019 Jul 16;7:497. doi: 10.3389/fchem.2019.00497 (PMC6646719; doi:10.3389/fchem.2019.00497)
Supplement: Supplementary file 1 [file Table_1.DOCX]

Fig. S1 SEM images of 1Cu-MBGN show the sphere-like shape and mesoporous surface of the particles.


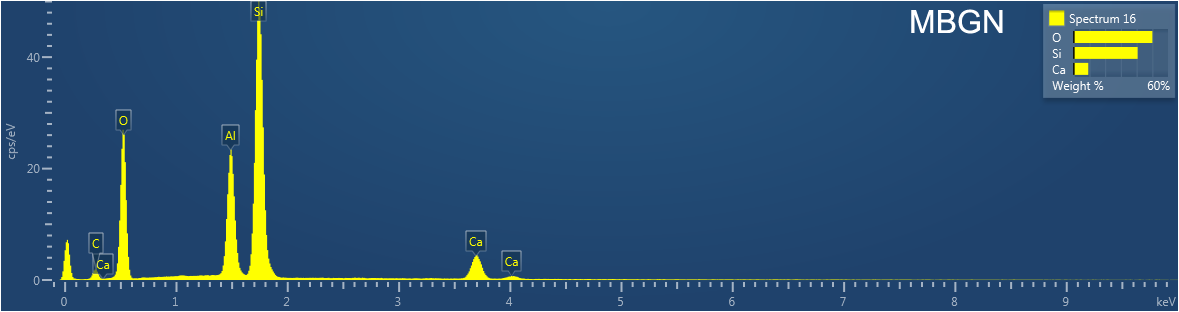

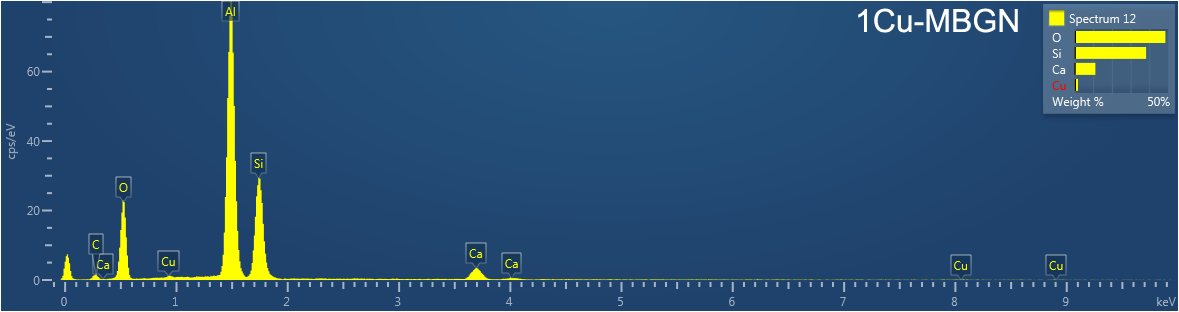

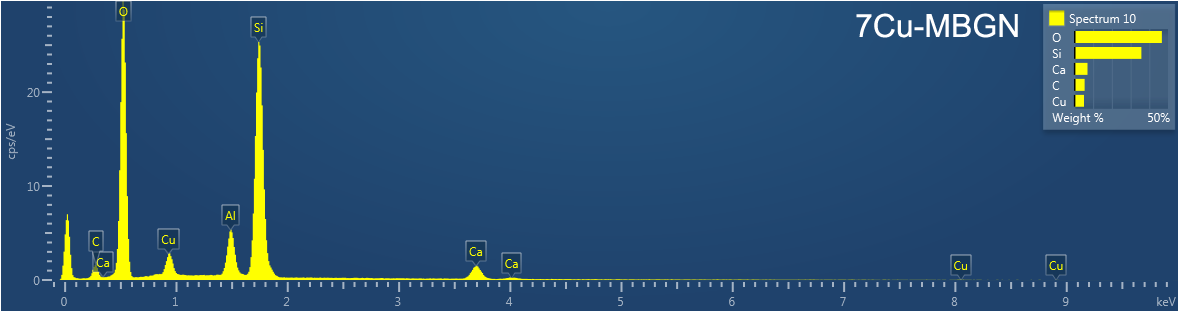

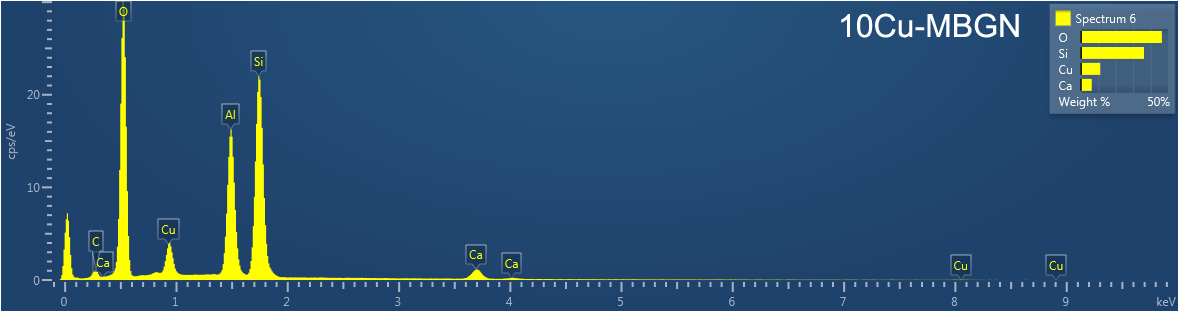


Fig. S2 EDS spectra of MBGN, 1Cu-MBGN, 7Cu-MBGN and 10Cu-MBGN show the presence of Si and Ca in MBGN and Si, Ca and Cu in Cu-MBGNs.









Fig. S3 SEM images of MBGN, 1Cu-MBGN, 7Cu-MBGN after immersion in SBF for 7d show the presence of needle-like HA crystals.









Fig. S4 Percentage of released ions in Tris-HCl for up to 7 d in relation to the chemical composition (determined by ICP-AES) of MBGN and Cu-MBGNs.
